# Correction to: micronutrient deficiency conditions: Global Health issues

**DOI:** 10.1186/s40985-017-0071-6

**Published:** 2017-10-31

**Authors:** Theodore H. Tulchinsky

**Affiliations:** Braun School of Public Health and Community Medicine, Hebrew University-Hadassah, Hadassah Ein Karem, Jerusalem, Israel

## Erratum

In the original publication of this article [1] Table 1 contained an error in 1 cell entry. In this correction the correct and incorrect cell are shown. The full table is available in the original article [1].

**Table 1:** original cell entry as published on 3 June 2010

Micronutrient	Deficiency Prevalence	Major Deficiency Disorders
Folate (Vitamin B6)	Insufficient data	Megaloblastic anemia, neural tube and other birth defects, heart disease, stroke, impaired cognitive function, depression

**Table 2:** Correct cell entry, “Vitamin B6” has been adjusted to “Vitamin B9”

Micronutrient	Deficiency Prevalence	Major Deficiency Disorders
Folate (Vitamin B9)	Insufficient data	Neural tube and other birth defects, megaloblastic anemia, heart disease, stroke, impaired cognitive function, depression
